# Baricitinib Treatment in RNU7-1-Associated Aicardi–Goutières Syndrome in a South African Child: A Case Report

**DOI:** 10.1002/ajmg.a.63978

**Published:** 2025-01-02

**Authors:** Timothy F. Spracklen, Shehnaaz Akhalwaya, Sally Ackermann, Carolina Uggenti, Luis Seabra, Yanick J. Crow, Kate Webb

**Affiliations:** 1 Department of Paediatrics and Child Health, University of Cape Town, Cape Town, South Africa; 2 Cape Heart Institute, University of Cape Town, Cape Town, South Africa; 3 Cape Paediatric Rheumatology, Cape Town, South Africa; 4 Mediclinic Constantiaberg, Cape Town, South Africa; 5 MRC Human Genetics Unit, Institute of Genetics and Cancer, the University of Edinburgh, Edinburgh, UK; 6 Laboratory of Neurogenetics and Neuroinflammation, University of Paris, Imagine Institute, Paris, France; 7 Crick African Network, the Francis Crick Institute, London, UK

**Keywords:** Aicardi–Goutières syndrome, baricitinib, JAK inhibitors, RNU7-1

## Abstract

Aicardi–Goutières syndrome (AGS) is a rare monogenic type I interferonopathy. Janus kinase (JAK) inhibition has emerged as a potential treatment for AGS. *RNU7-1* is one of the most recently discovered genes for AGS, and the clinical effects of JAK in-hibition in these patients have not been reported. Here, we describe the diagnosis and treatment of a South African infant with *RNU7-1*-related AGS. The patient presented with developmental delay at age 5 months and was diagnosed with cerebral palsy due to a suspected congenital infection. By 18 months of age, he had a vasculitic rash, prominent generalized dystonia, persistent transaminitis, recurrent stomatitis, moderate-range global developmental delay, and difficulty sleeping. AGS was considered after finding neuroimaging features of the disease; the diagnosis was confirmed when genetic investigations revealed two likely pathogenic *RNU7-1* compound heterozygous variants in the patient. Elevated interferon gene expression was noted in the patient and his mother who was a carrier of one *RNU7-1* variant. Baricitinib treatment was started, leading to modest, transient improvements in some clinical manifestations and a reduction in interferon-stimulated gene expression. Liver function, dystonia, and neurological function did not improve even after increasing the baricitinib dose. Baricitinib was discontinued due to persistent and worsening adverse effects.

## 1 Introduction

Aicardi–Goutières syndrome (AGS) is a rare autoinflammatory monogenic disorder and type I interferonopathy. Although signs and symptoms may vary in presentation and severity, typical features of AGS include early-onset encephalopathy (within the first year of life), and other neurological features such as irritability, microcephaly, epileptic seizures, developmental delay, intracranial calcification, and motor disorder (Crow and Manel 2015). As a type I interferonopathy, patients with AGS usually exhibit increased type I interferon (IFN-I) signaling in blood and cerebrospinal fluid (Rice et al. 2013). AGS may affect other organs, leading to liver and kidney disease as well as skin manifestations. Nine AGS-related genes have been described to date (Liu and Ying 2023). These genes are involved in nucleic acid processing and sensing, and pathogenic variants lead to IFN-I overexpression and autoinflammation. *RNU7-1* is one of the most recently discovered genes for AGS (Uggenti et al. 2020; Naesens et al. 2022), annotated as AGS9 and accounting for less than 1% of AGS cases to date (Liu and Ying 2023).

While there is currently no cure for type I interferonopathies, potential treatments might involve targeting IFN-I signaling (with Janus kinase [JAK] inhibitors) or reverse transcription of endogenous retroelements (with reverse-transcriptase inhibitors) (Cetin Gedik et al. 2022; Dell’Isola et al. 2023). Although not officially approved and with relatively limited evidence of their clinical efficacy, the use of JAK inhibitors tofacitinib, ruxolitinib, and baricitinib has been described in case reports and series as potentially effective in ameliorating certain features of AGS. As one of the most recently described AGS genes, the effect of JAK inhibition in *RNU7-1*-related AGS is less established. However, in the initial report describing variants in *RNU7-1* as a cause of AGS, patient fibroblasts demonstrated a reduction in *IFIT1* expression upon treatment with ruxolitinib (Uggenti et al. 2020), indicating the potential for treating such patients with JAK inhibitors. However, more data are needed to determine whether these patients will respond as favorably to JAK inhibition as other forms of AGS.

Here, we describe a South African child with AGS due to *RNU7-1* pathogenic variants and his treatment with baricitinib.

## 2 Case Report

A 5-month-old male infant initially presented with global developmental delay. He was the first child of non-consanguineous parents with no previous pregnancy losses and no relevant family history. No perinatal risk factors were identified. He developed irritability at age 6 weeks, which was attributed to reflux. There was no associated developmental regression. On examination, he had central hypotonia, increased limb tone, pyramidal tract signs, and dystonia of all limbs. Head circumference plotted on the mean z-score for age based on World Health Organization growth standards. He displayed a non-paralytic convergent strabismus and nystagmus. Retinal examination was normal, and he was diagnosed with cortical visual impairment. Audiological assessment revealed normal hearing. A ventricular septal defect was detected postnatally and closed spontaneously. Computed tomography imaging of the brain revealed the presence of multiple nodular calcifications in the deep white matter, gray-white matter interface, and basal ganglia; magnetic resonance imaging confirmed these findings. Screening for congenital infections was negative although urinary cytomegalovirus polymerase chain reaction (PCR) was not performed. Metabolic workup was limited to serum amino acids (normal). He was diagnosed with cerebral palsy due to a suspected congenital infection.

**Figure 1:**
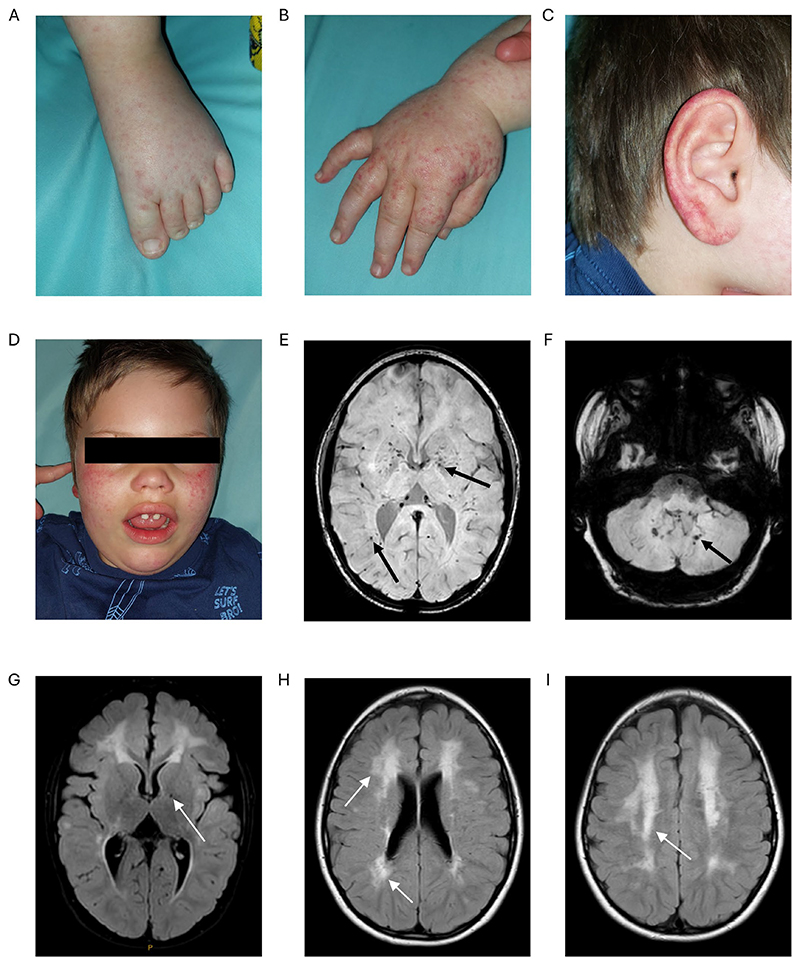
Clinical features of the AGS patient. (A-D) Vasculitic rash over the extremities, which included fingers, feet, ears, and cheeks with mild soft-tissue edema; photographs were taken at age five years, one month before baricitinib treatment started in 2022. Post-treatment photographs were not available. (E, F) Axial susceptibility weighted imaging at age 7 years, one year after baricitinib, demonstrating multifocal, symmetrical areas of blooming artifact in the basal ganglia, and deep white matter of the cerebral hemispheres (E) and dentate nuclei within the cerebellum (F) (black arrows). (G-I) Axial FLAIR imaging at age 7 years demonstrating confluent symmetrical hyperintensity in the periventricular and deep white matter of both cerebral hemispheres (white arrows).

At age 18 months the family relocated, by which time the patient’s head growth had continued along the mean z-score and his vision had significantly improved. He had a vasculitic rash over the extremities including the fingers, feet, ears, and cheeks with mild soft-tissue non-pitting edema (Figure 1A–D). He had developed recurrent stomatitis. Neurologically, he had prominent generalized dystonia requiring regular intramuscular botulinum toxin injections. He had difficulty sleeping and moderate-range global developmental delay with better-developed receptive language. He had a mildly elevated urinary protein:creatinine ratio but no renal impairment, pericardial effusion, Raynaud’s syndrome, glaucoma, or bone involvement. Liver function tests revealed a persistent transaminitis. Inflammatory markers remained normal. Repeat neuroimaging revealed prominent basal ganglia and periventricular calcification, delayed myelination, and extensive white matter signal alterations (Figure 1F–I). A diagnosis of AGS was considered, and he was referred to pediatric rheumatology.

Genetic investigation by whole genome sequencing revealed two *RNU7-1* variants in the patient, consistent with a diagnosis of AGS9: n.28C > A (paternally inherited) and n.54G > A (maternally inherited). These variants fulfilled ACMG criteria for likely pathogenicity (Richards et al. 2015) due to: 1. Their absence (n.28C > A) or extremely low frequency (n.54G > A) in population genomic databases [PM2]; 2. The observation of similar substitutions at the same nucleotides, which have been determined to be pathogenic in other AGS patients (Uggenti et al. 2020; Naesens et al. 2022) [PM5]; 3. The phenotype of the patient is consistent with a monogenic disease [PP4]; and 4. These variants occur in critical regions and well-established functional domains of the *RNU7-1* gene product (Naesens et al. 2022) [PM1].

The patient was then managed symptomatically. His skin features were particularly troublesome having flared multiple times; therefore, treatment with a JAK inhibitor was considered. He was started on 0.2 mg/kg baricitinib per day, and initially there appeared to be an appreciable clinical improvement in his disease; his parents reported an improvement in skin manifestations and he had no further episodes of stomatitis. His sleep and irritability intermittently improved for the first month before returning to baseline. Liver function tests did not show a consistent improvement, and there was no improvement in dystonia. He experienced some manageable adverse effects, including nausea and vomiting.

Due to the modest improvement in his clinical condition, after 6 months the dose was increased to 0.25 mg/kg baricitinib per day. After 5 months on the increased dose, neurological signs and liver function tests remained static, and the early apparent skin improvement was not maintained. Baricitinib treatment was discontinued due to persistent and worsening nausea and headaches, combined with the lack of an appreciable improvement in the patient’s clinical status or laboratory results. Two months after baricitinib treatment was discontinued, he developed arthritis in his left knee requiring intra-articular steroid injection. He has since developed migraine-like headaches, but at age 7 years his neurological phenotype remains unchanged, and there has been no further disease progression. He remains nonverbal, is able to sit independently with good head control, but has no independent mobility (GMFCS level IV). He has limited functional use of his upper limbs and good nonverbal communication skills. He attends an alternative learning program class at a school catering for children with physical disabilities.

**Figure 2:**
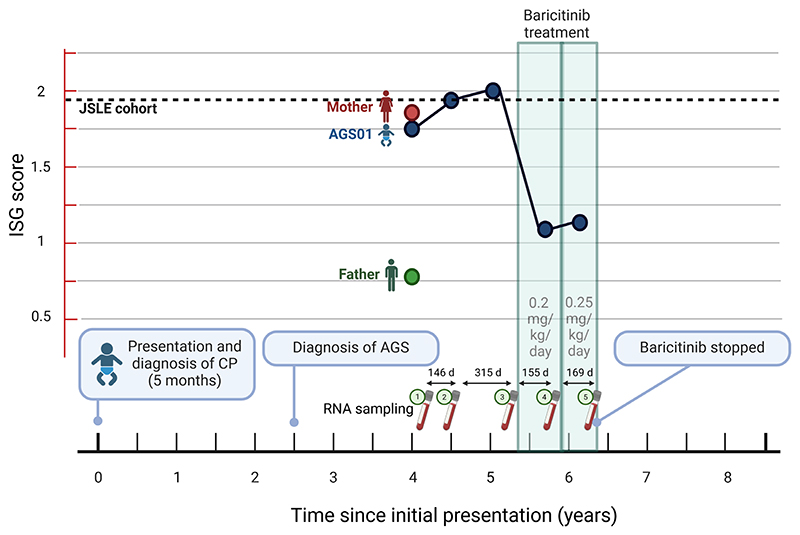
Timeline of the AGS case from presentation to diagnosis and baricitinib treatment. Indicated are the RNA sampling times and resulting interferon-stimulated gene (ISG) score in the patient (AGS01, blue), his mother (red), and his father (green). The dashed line indicates the median ISG score derived from a cohort of treatment-naïve patients with juvenile lupus erythematosus (JSLE).

Whole blood RNA samples were taken at various timepoints throughout the patient’s clinical course, including post-treatment samples after the initiation of 0.2 mg/kg baricitinib per day and after the dosage was increased to 0.25 mg/kg per day (Figure 2). These samples were analyzed by multiplex quantitative PCR as previously described (Spracklen et al. 2022). We developed a panel of six IFN-I stimulated genes (ISGs) (*DDX58, IRF7, ISG15, MX1, MX2, OAS1*), which were all raised based on the fold change in a cohort of 12 treatment-naïve children with juvenile systemic lupus erythematosus (JSLE), which is associated with enhanced type 1 ISG expression, compared to 74 healthy children (Supplementary figure 1). Using the mean fold changes of these genes relative to healthy children as an ISG score, all were similarly overexpressed in the AGS patient as they were in the JSLE cohort, except for *IRF7*. We observed a decrease in ISG expression during treatment with baricitinib (Figure 2). Expression of all genes reduced after baricitinib treatment started and reduced further after the dosage was increased. Notably, the patient’s mother, who carried the *RNU7-1* n.54G > A variant, had elevated expression of IFN-I genes, especially *DDX58*, which was higher than the AGS patient and the JSLE cohort.

## 3 Discussion

In this South African patient with AGS due to biallelic *RNU7-1* pathogenic variation, JAK inhibition was associated with a decreased expression of ISGs but had limited clinical effect. This is an example of a rare cause of AGS, with only 19 cases of *RNU7-1*-related AGS described to date. Although we did not investigate for a disturbance of histone mRNAs or histone stoichiometry, the genetics and clinical data in our patient are consistent with AGS due to pathogenic *RNU7-1* variation. Of the 19 other AGS9 patients in the literature (Naesens et al. 2022), all had neurological involvement, developmental delay, and hypertonia or spasticity, as seen in our patient. Intracranial calcification and other neuroimaging abnormalities were also reported as common features of AGS9. Liver disease, skin manifestations, and irritability were other frequent signs also present in our patient. Cardiovascular involvement is also relatively common, with 29% reporting pericardial effusions, and our patient being the first, to our knowledge, with a spontaneously resolving ventricular septal defect.

JAK inhibition has been considered for the treatment of type I interferonopathies such as AGS. A clinical trial of baricitinib in the largest cohort to date, consisting of 35 patients with genetically confirmed AGS, demonstrated improvement in neurologic function, liver function, and developmental skills, as well as a reduction in an ISG expression score during treatment (Vanderver et al. 2020). In that study, the JAK inhibitor was overall well tolerated, although risks included thrombocytosis, leukopenia, and infection (Vanderver et al. 2020). Other isolated reports have confirmed the beneficial effects of JAK inhibition in the context of AGS (Sanchez et al. 2018; Kanazawa et al. 2023; Ryckmans et al. 2024; Panigrahy et al. 2022), in some cases apparently leading to complete recovery of cognitive and fine motor skills (Galli et al. 2023). Other benefits include reduction of chilblains (Meesilpavikkai et al. 2019), pericardial effusion (Casas-Alba et al. 2022), and IFN gene expression (Sanchez et al. 2018; Kanazawa et al. 2023; Ryckmans et al. 2024; Galli et al. 2023; Meesilpavikkai et al. 2019; Casas-Alba et al. 2022). Similarly, treatment with ruxolitinib or tofacitinib has, in most, cases led to improvement in neurological symptoms while alleviating systemic features such as chilblains, stroke, lung disease, and renal disease (Cattalini et al. 2021; Kuang et al. 2022; Li et al. 2022; Tüngler et al. 2016; Kothur et al. 2018; Mura et al. 2021; Pararajasingam et al. 2022; Wang et al. 2023; Sorokina, Raupov, and Kostik 2023; Zhang et al. 2021; Zheng et al. 2020) and markedly reducing IFN gene expression (Cattalini et al. 2021; Tüngler et al. 2016; Mura et al. 2021).

The efficacy of JAK inhibition in AGS is not conclusive; however, some cases report no benefit and severe progression of neurological disease, even with pre-emptive treatment (Železnik et al. 2022; Jafarpour et al. 2024; Neven et al. 2020). In our patient, we saw limited benefits of baricitinib, although this could be attributed to the relatively low dose. There was no improvement in neurological disease or liver function and only transient improvement in sleep disturbance, irritability, and rash despite uninterrupted treatment. We did, however, see a reduction in ISG expression over the study period. In a long-term assessment of 11 AGS patients, JAK inhibition had very limited benefits on neurological manifestations of disease after a median of 17 months, despite improvements in skin lesions, other systemic features, and ISG scores (Frémond et al. 2023). Treatment with other JAK inhibitors such as ruxolitinib or tofacitinib has produced similarly variable results, despite a reduction in ISG expression (Cattalini et al. 2021; Tüngler et al. 2016; Mura et al. 2021). It is likely that limited penetration of the central nervous system by JAK inhibitors underlies these observed systemic benefits with variable effects on neurological or cognitive outcomes (Neven et al. 2020).

## 4 Conclusion

This case highlights a rare genetic cause of AGS and expands on the clinical presentation of *RNU7-1* pathogenic variants. Although JAK inhibition has shown promise as a treatment in other genetic forms of AGS, our experience in treating this patient with baricitinib demonstrates that further studies of the effectiveness of JAK inhibitors are needed in the context of *RNU7-1*-related AGS. We also show that ISG expression may be elevated in otherwise healthy parents of AGS patients; the implications of this will need to be explored further.

## Supplementary Material

Supplementary Material

## Data Availability

Data sharing is not applicable to this article as no new data were created or analyzed in this study.

## References

[R1] Casas-Alba D, Darling A, Caballero E (2022). Efficacy of Baricitinib on Chronic Pericardial Effusion in a Patient With Aicardi-Goutières Syndrome. Rheumatology (Oxford, England).

[R2] Cattalini M, Galli J, Zunica F (2021). Case Report: The JAK-Inhibitor Ruxolitinib Use in Aicardi-Goutieres Syndrome due to ADAR1 Mutation. Frontiers in Pediatrics.

[R3] Cetin Gedik K, Lamot L, Romano M (2022). The 2021 European Alliance of Associations for Rheumatology/American College of Rheumatology Points to Consider for Diagnosis and Management of Autoinflammatory Type I Interferonopathies: CANDLE/PRAAS SAVI and AGS. Ann Rheum Dis.

[R4] Crow YJ, Manel N (2015). Aicardi-Goutières Syndrome and the Type I Interferonopathies. Nature Reviews Immunology.

[R5] Dell’Isola GB, Dini G, Culpepper KL (2023). Clinical Spectrum and Currently Available Treatment of Type I Interferonopathy Aicardi-Goutières Syndrome. World Journal of Pediatrics.

[R6] Frémond ML, Hully M, Fournier B (2023). JAK Inhibition in Aicardi-Goutières Syndrome: A Monocentric Multidisciplinary Real-World Approach Study. Journal of Clinical Immunology.

[R7] Galli J, Cattalini M, Loi E (2023). Treatment Response to Janus Kinase Inhibitor in a Child Affected by Aicardi-Goutières Syndrome. Clinical Case Reports.

[R8] Jafarpour S, Suddock J, Hawes D, Santoro JD (2024). Neuropathologic Impacts of JAK Inhibitor Treatment in Aicardi-Goutières Syndrome. Journal of Clinical Immunology.

[R9] Kanazawa N, Ishii T, Takita Y, Nishikawa A, Nishikomori R (2023). Efficacy and Safety of Baricitinib in Japanese Patients With Autoinflammatory Type I Interferonopathies (NNS/CANDLE, SAVI, and AGS). Pediatric Rheumatology Online Journal.

[R10] Kothur K, Bandodkar S, Chu S (2018). An Open-Label Trial of JAK 1/2 Blockade in Progressive IFIH1-Associated Neuroinflammation. Neurology.

[R11] Kuang SY, Li Y, Yang SL, Han X (2022). Child Neurology: Aicardi-Goutières Syndrome Presenting as Recurrent Ischemic Stroke. Neurology.

[R12] Li W, Wang W, Wang W (2022). Janus Kinase Inhibitors in the Treatment of Type I Interferonopathies: A Case Series From a Single Center in China. Frontiers in Immunology.

[R13] Liu A, Ying S (2023). Aicardi-Goutières Syndrome: A Monogenic Type I Interferonopathy. Scandinavian Journal of Immunology.

[R14] Meesilpavikkai K, Dik WA, Schrijver B (2019). Efficacy of Baricitinib in the Treatment of Chilblains Associated With Aicardi-Goutières Syndrome, a Type I Interferonopathy. Arthritis & Rhematology.

[R15] Mura E, Masnada S, Antonello C (2021). Ruxolitinib in Aicardi-Goutières syndrome. Metabolic Brain Disease.

[R16] Naesens L, Nemegeer J, Roelens F (2022). Mutations in RNU7-1 Weaken Secondary RNA Structure, Induce MCP-1 and CXCL10 in CSF, and Result in Aicardi-Goutières Syndrome With Severe End-Organ Involvement. Journal of Clinical Immunology.

[R17] Neven B, Al Adba B, Hully M (2020). JAK Inhibition in the Aicardi-Goutières Syndrome. New England Journal of Medicine.

[R18] Panigrahy N, Bakhru S, Lingappa L, Chirla D (2022). Aicardi-Goutières Syndrome (AGS): Recurrent Fetal Cardiomyopathy and Pseudo-TORCH Syndrome. BML Case Reports.

[R19] Pararajasingam A, Bradley RE, Evans J, Lowe A, Goodwin R, Jolles S (2022). Case Report: Generalised Panniculitis as a Post-COVID-19 Presentation in Aicardi-Goutières Syndrome Treated With Ruxolitinib. Frontiers in Pediatrics.

[R20] Rice GI, Forte GMA, Szynkiewicz M (2013). Assessment of Interferon-Related Biomarkers in Aicardi-Goutières Syndrome Associated With Mutations in TREX1, RNASEH2A, RNASEH2B, RNASEH2C, SAMHD1, and ADAR: A Case–Control Study. Lancet Neurology.

[R21] Richards S, Aziz N, Bale S (2015). Standards and Guidelines for the Interpretation of Sequence Variants: A Joint Consensus Recommendation of the American College of Medical Genetics and Genomics and the Association for Molecular Pathology. Genetics in Medicine.

[R22] Ryckmans C, Donge M, Marchese A (2024). TREX-1 Related Aicardi-Goutières Syndrome Improved by Janus Kinase Inhibitor. American Journal of Medical Genetics Part A.

[R23] Sanchez GAM, Reinhardt A, Ramsey S (2018). JAK1/2 Inhibition With Baricitinib in the Treatment of Autoinflammatory Interferonopathies. Journal of Clinical Investigation.

[R24] Sorokina LS, Raupov RK, Kostik MM (2023). Juvenile Dermatomyositis and Infantile Cerebral Palsy: Aicardi-Gouteres Syndrome, Type 5, With a Novel Mutation in SAMHD1-A Case Report. Biomedicine.

[R25] Spracklen TF, Mendelsohn SC, Butters C (2022). IL27 Gene Expression Distinguishes Multisystem Inflammatory Syndrome in Children From Febrile Illness in a South African Cohort. Frontiers in Immunology.

[R26] Tüngler V, König N, Günther C (2016). Response to: ‘JAK Inhibition in STING-Associated Interferonopathy’ by Crow Et al. Annals of the Rheumatic Diseases.

[R27] Uggenti C, Lepelley A, Depp M (2020). cGAS-Mediated Induction of Type I Interferon due to Inborn Errors of Histone Pre-mRNA Processing. Nature Genetics.

[R28] Vanderver A, Adang L, Gavazzi F (2020). Janus Kinase Inhibition in the Aicardi-Goutières Syndrome. New England Journal of Medicine.

[R29] Wang W, Wang W, Peng S (2023). Tocilizumab Reduces the Unmanageable Inflammatory Reaction of a Patient With Aicardi-Goutières Syndrome Type 7 During Treatment With Ruxolitinib. Pediatric Rheumatology Online Journal.

[R30] Železnik M, Soltirovska Salamon A, Debeljak M (2022). Case Report: Pneumocystis Jirovecii Pneumonia in a Severe Case of Aicardi-Goutières Syndrome With an IFIH1 Gain-Of-Function Mutation Mimicking Combined Immunodeficiency. Frontiers in Immunology.

[R31] Zhang S, Song J, Yang Y (2021). Type I Interferonopathies With Novel Compound Heterozygous TREX1 Mutations in Two Siblings With Different Symptoms Responded to Tofacitinib. Pediatric Rheumatology Online Journal.

[R32] Zheng S, Lee PY, Wang J (2020). Interstitial Lung Disease and Psoriasis in a Child With Aicardi-Goutières Syndrome. Frontiers in Immunology.

