# Supplementary material for: Baricitinib Treatment in RNU7-1-Associated Aicardi–Goutières Syndrome in a South African Child: A Case Report
Source: Am J Med Genet A. Author manuscript; Available in PMC 2025 May 1. (PMC7617338; doi:10.1002/ajmg.a.63978)
Supplement: Supplementary Material [file EMS202145-supplement-Supplementary_Material.pdf]

Supplementary figure 1

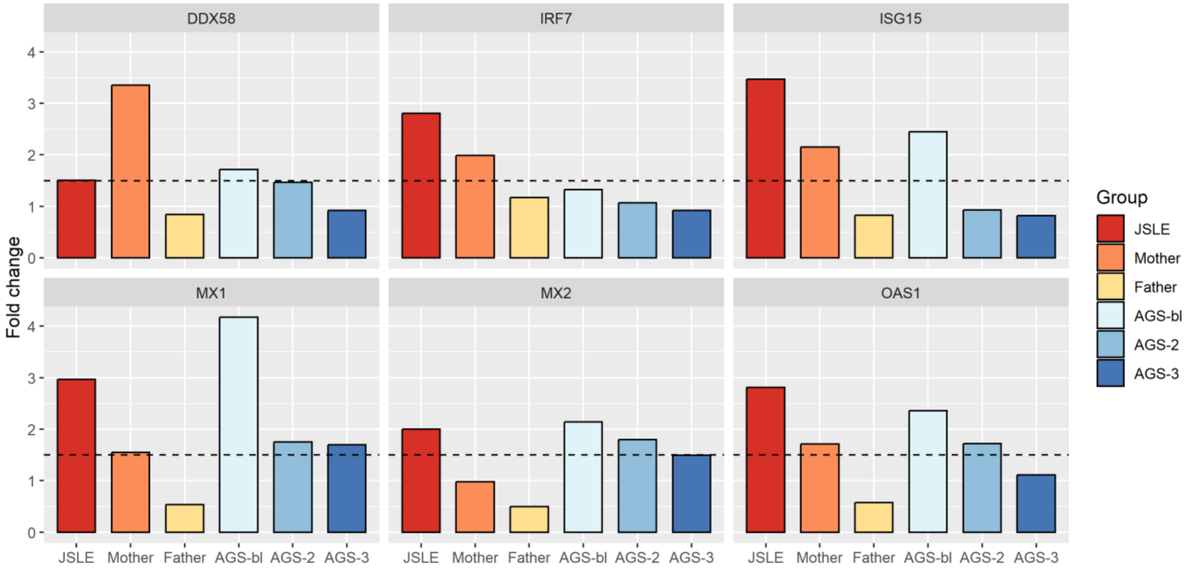

Supplementary figure 1: IFN-I stimulated gene up-regulation in the AGS patient compared to children with juvenile systemic lupus erythematosus. The AGS patient (blue bars) was sampled three times before treatment, with the average illustrated here (AGS-bl). He was sampled again after 4 mg baricitinib (AGS-2) and after 6 mg baricitinib (AGS-3). The patient's mother (orange) and father (yellow) were both sampled at the first pre-treatment timepoint for the child with AGS. The dashed line indicates 1.5-fold up-regulation of gene expression.
